# Associations of early childhood caries and child intelligence quotient: evidence from the Shanghai Birth Cohort

**DOI:** 10.3389/fpubh.2026.1803559

**Published:** 2026-06-01

**Authors:** Chuchu Wang, Qian Chen, Hui Wang, Huning Wang, Xiaoli Zeng, Kaixuan Tang, Liuyang Xu, Jun Zhang, Ying Zhang, Jianfeng Luo, Hao Zhang

**Affiliations:** 1 Department of Biostatistics, School of Public Health, Fudan University, Shanghai, China; 2 Department of Preventive Dentistry, Shanghai Stomatological Hospital & School of Stomatology, Fudan University, Shanghai, China; 3 NHC Key Laboratory of Health Technology Assessment, Fudan University, Shanghai, China; 4 Key Laboratory of Public Health Safety of Ministry of Education, Fudan University, Shanghai, China; 5 Ministry of Education-Shanghai Key Laboratory of Children’s Environmental Health, Xinhua Hospital, Shanghai Jiao Tong University School of Medicine, Shanghai, China; 6 Shanghai Key Laboratory of Craniomaxillofacial Development and Diseases, Fudan University, Shanghai, China

**Keywords:** cognition, dental caries, early childhood, intelligence quotient, oral health

## Abstract

**Introduction and aims:**

Growing evidence indicates that early childhood oral health, particularly dental caries, may be associated with children’s cognitive development; however, findings remain inconsistent, especially in preschool-aged populations. This study aimed to examine the associations between early childhood caries and intelligence quotient (IQ) in four-year-old children.

**Method:**

This study used a cross-sectional design based on data from 1,099 four-year-old children in the Shanghai Birth Cohort. Oral health measurements included the decayed, missing, filled teeth (dmft) index, and caries status. Child IQ was assessed using the fourth edition of the Wechsler Preschool and Primary Scale of Intelligence (WPPSI-IV). Multivariable linear regression models were used to estimate the associations between oral health indicators and child IQ. Restricted cubic spline models were used to assess non-linear relationships.

**Results:**

Higher dmft index scores were significantly associated with lower full scale intelligence quotient (FSIQ) scores (*β* = −0.25; 95% CI: −0.48, −0.03) and verbal comprehension index (VCI) scores (*β* = −0.31; 95% CI: −0.56, −0.05) after adjusting for potential confounders. Consistently, children with caries showed significantly lower FSIQ scores than those without caries (*β* = −1.59; 95% CI: −3.01, −0.18).

**Conclusion:**

Dental caries, particularly as measured by the dmft index, was associated with lower IQ scores in preschool children. These findings should be interpreted as population-level associations rather than deterministic outcomes for individual children. Longitudinal studies are needed to confirm these findings and clarify the mechanisms linking early oral health and neurocognitive development.

## 1 Introduction

Early childhood caries (ECC) is the presence of one or more decayed (noncavitated or cavitated lesions), missing (because of caries), or filled tooth surfaces in any primary tooth in a child aged 72 months or younger (1). Beyond its direct dental implications, ECC can compromise a child’s overall health and well-being. Although preventable, ECC remains a serious oral health problem on a global scale (2, 3), leading to negative impacts on children’s oral function, nutrition, quality of life, and developmental trajectory (4–6). ECC may affect cognitive development through several interrelated pathways. Caries-related pain, infection, impaired mastication, sleep disturbance, and nutritional problems may influence children’s growth, attention, learning ability, and neurodevelopment (7–19). Collectively, these pathways suggest that oral health problems may influence cognitive outcomes through both neurophysiological and behavioral mechanisms. Notably, the preschool years constitute a sensitive period for neurodevelopment, during which the brain shows high plasticity and rapid advances in information processing. At this stage, children also acquire a wide range of abilities essential for school adaptation (20–22). Core cognitive processes—including perception, recognition, executive control, reasoning, and memory—are regarded as academic-related factors that have lasting implications for health and psychosocial outcomes. Evidence further indicates that cognitive performance in early childhood (approximately 2–6 years) is predictive of subsequent academic achievement, both in the near term and throughout later schooling (23, 24).

Despite the recognized importance of this developmental stage, most existing studies have focused on school-aged children, examining associations between oral health and academic outcomes (25–27). Prior evidence indicates that poorer oral health—particularly dental caries and toothache—is linked to reduced academic performance, psychosocial difficulties, and increased school absenteeism (28–32). However, some studies have yielded inconsistent results, reporting either negative (33–35) or null associations (28, 36). Although existing evidence highlights the potential influence of oral health on learning and behavior, little is known about whether these associations emerge during the preschool period, before formal schooling begins. In contrast, studies investigating oral health in relation to cognitive development among preschoolers remain scarce, with most focusing on developmental delays or neurodevelopmental disorders rather than typical cognitive performance (37, 38). Thus, we aimed to examine the association between ECC and child IQ at age four. We hypothesize that higher ECC burden is associated with lower IQ in preschool children.

## 2 Materials and methods

### 2.1 Study population

**Figure 1:**
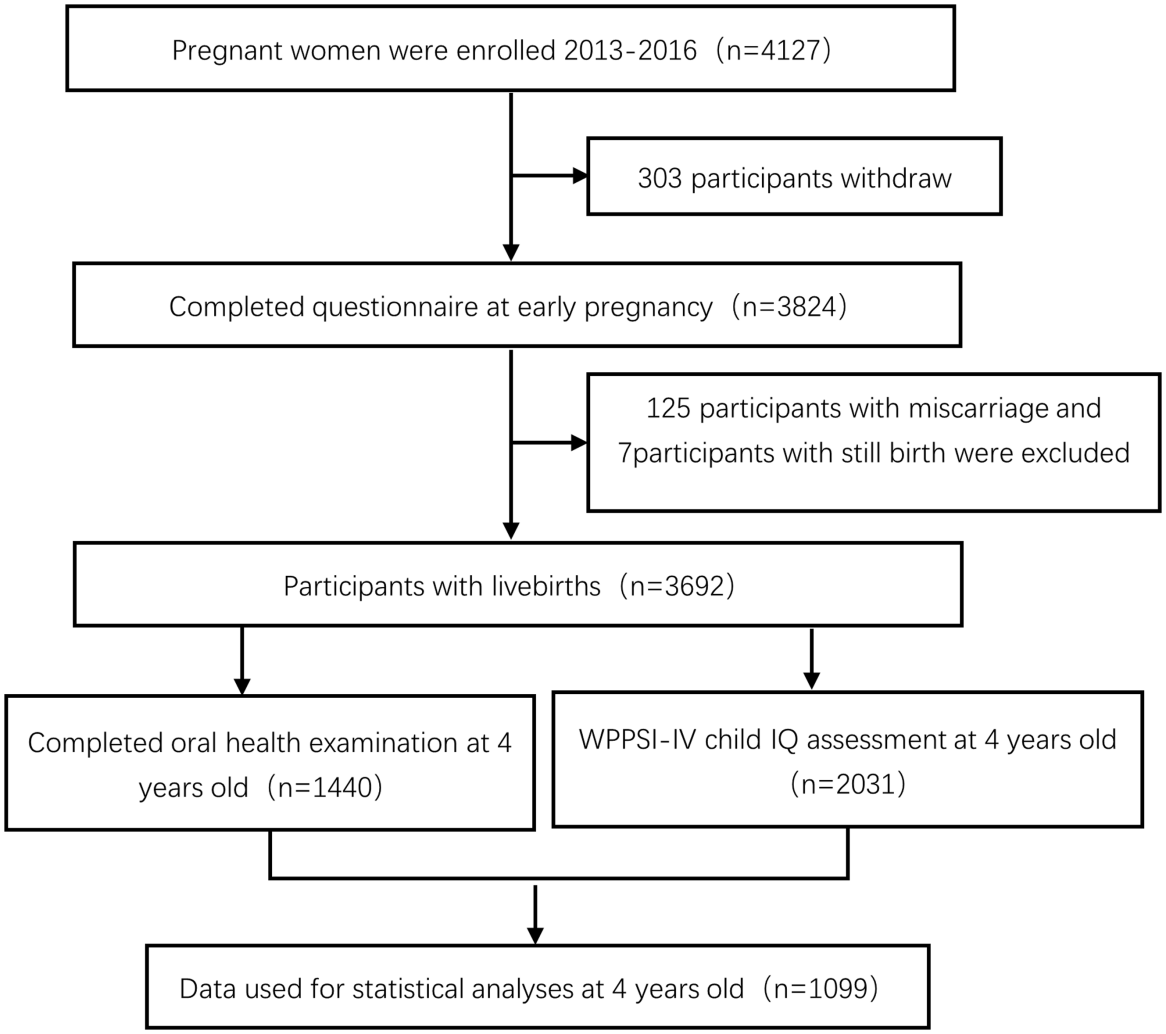
Participant recruitment flowchart.

This cross-sectional study, based on the Shanghai Birth Cohort (SBC), recruited 3,692 mother–child pairs between 2013 and 2016 from women enrolled during pre-pregnancy and early pregnancy at six hospitals across four administrative districts in Shanghai, China (39). At the study entry, the following information including demographic characteristics (e.g., child’s age in months, gender, maternal age, maternal education level, and annual household income), child lifestyle and health-related factors (e.g., passive smoking duration per day in the past month and body mass index at age four), and early life characters (e.g., breastfeeding status, preterm birth, and birth weight) were collected through self-administered questionnaires. According to the original study design, a subset of cohort children was selected and invited to participate in a detailed follow-up assessment at 4 years of age. From 2017 to 2020, the selected families were contacted and invited to attend a face-to-face follow-up visit, which included an oral health examination, an intelligence quotient (IQ) assessment of the child, and a diagnostic interview with the primary caregiver, usually the mother. Among the children participating in the 4-year follow-up, 2,031 completed the IQ assessment and 1,440 completed the oral health examination. Finally, 1,099 children with both oral health examination data and IQ assessment data were included in the present statistical analyses. The flow of participants throughout the study is illustrated in Figure 1.

The study was approved by the ethics committees of Xinhua Hospital affiliated to the Shanghai Jiao Tong University. All participants signed the informed consent prior to their enrollment.

### 2.2 Dental caries measurement

The evaluation of Oral health status in 4-year-old children was conducted by trained and calibrated examiners from Shanghai Stomatological Hospital. All assessments were performed under standardized conditions using white light from a head torch, a plane mouth mirror, and a smooth-ended probe to ensure diagnostic accuracy and consistency.

Dental caries was assessed according to the WHO criteria. A tooth was classified as carious if a lesion was present in a pit, fissure, or smooth surface with a visible cavity, undermined enamel, or softened floor or wall (40). Restored teeth and those missing due to caries were also recorded. The decayed, missing, and filled teeth (dmft) index was calculated by summing the number of decayed (d), missing (m), and filled (f) teeth, and was used to indicate caries prevalence and severity. Caries status was categorized using this index: a dmft score of 0 indicated caries-free, while a score >0 indicated caries presence.

### 2.3 Child IQ assessment

Child IQ at age 4 was assessed using the most recent edition of the Wechsler Preschool and Primary Scale of Intelligence (WPPSI-IV), a widely recognized and reliable instrument for evaluating cognitive development in young children (41). It has high-quality standards and high clinical validity internationally (42). The assessment includes 10 core subtests generating a full-scale IQ (FSIQ) and five primary index scores: verbal comprehension index (VCI), visual spatial index (VSI), fluid reasoning index (FRI), working memory index (WMI), and processing speed index (PSI) (43). IQ is a standardized index of intellectual ability that reflects performance across multiple cognitive domains (44). Trained neurodevelopmental pediatricians conducted the tests in a quiet, controlled environment following standardized procedures (45). Age-standardized scaled scores based on Chinese norms were used, with all indices having a population mean of 100 ± 15, where higher scores indicate better cognitive performance.

### 2.4 Covariates

**Figure 2:**
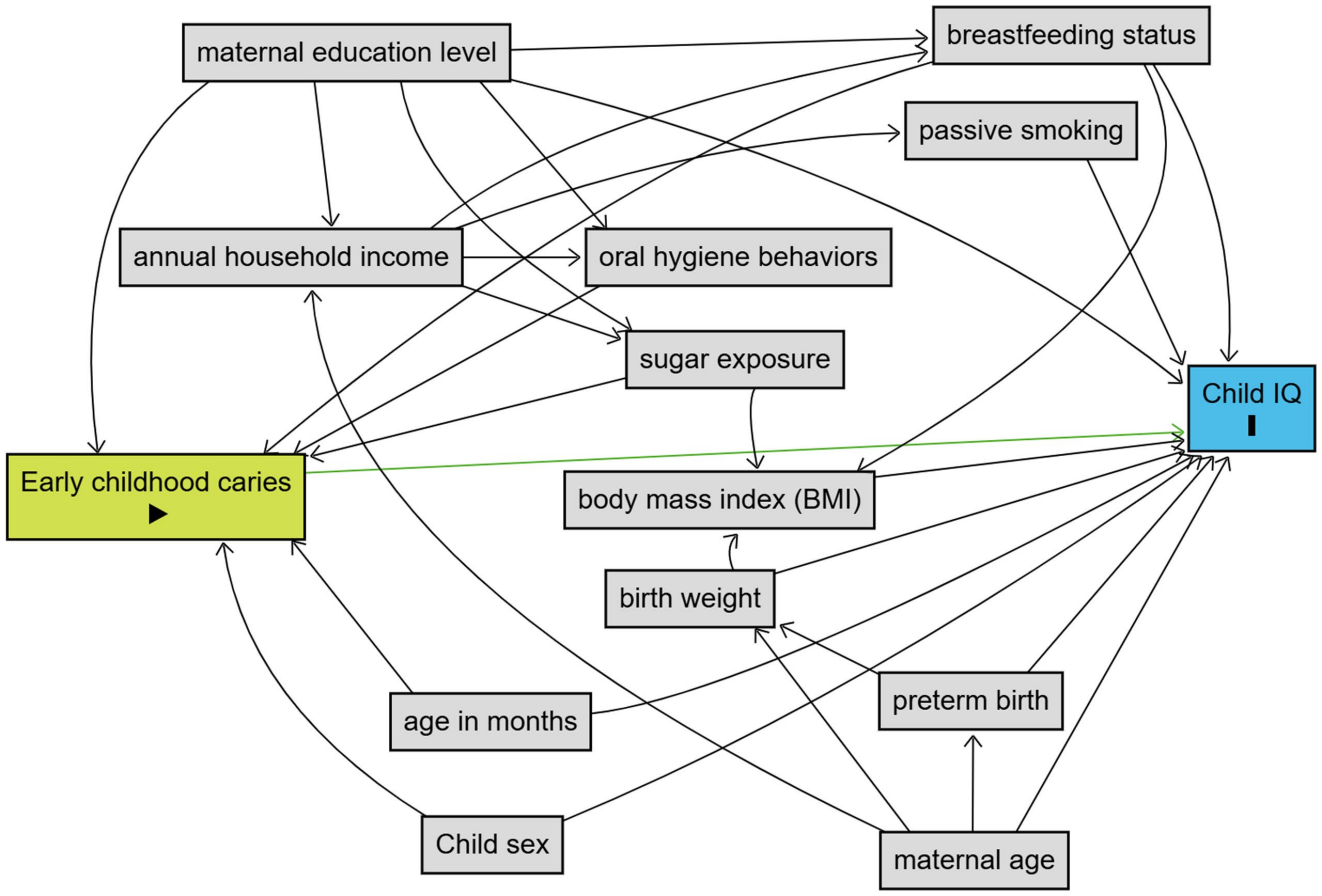
Directed acyclic graph for covariate selection.

Potential confounders were identified based on causal inference principles using Directed Acyclic Graphs (DAGs) constructed with the DAGitty tool.^1^ Information on covariates was collected through structured questionnaires completed by the participants’ mothers during the follow-up visits. As illustrated in Figure 2, the final analytical models included the following covariates: child’s age in months at IQ assessment, maternal age at delivery, birth weight, child’s body mass index (BMI) calculated using weight in kilograms divided by height in meters squared, child’s sex (male, female), maternal educational level (less than bachelor’s degree, bachelor’s degree, graduate degree or above), annual household income (<50,000; 50,000–99,999; 100,000–149,999; 150,000–299,999; ≥300,000 RMB), breastfeeding status (never; <6 months; 6–12 months; >12 months), passive smoking exposure in the past month (none; <1 h/day; 1–2 h/day; 3–5 h/day; >5 h/day), and preterm birth status (yes, no), dietary sugar intake including sweet pastries and candy/chocolate (≤1–3 times /month; 1–3 times /week; ≥4 times/week), sugar-sweetened beverage intake including sweetened fruit-flavored beverages and carbonated beverages (rarely or never; 1–3 times /month; ≥1 time /week), toothbrushing frequency (≤1 time /day; >1 time /day), and fluoride application frequency (never; once; ≥2 times). These covariates were included as potential confounders due to their associations with both oral health and cognitive outcomes.

### 2.5 Statistical analysis

We first conducted descriptive analyses to summarize the distributions of oral health indicators, covariates, and cognitive outcomes. Continuous variables, including IQ scores, birth weight, child age, maternal age, BMI, and the dmft index, were reported as mean ± standard deviation (SD), while categorical variables were presented as counts and percentages. Before regression modeling, missing data were handled using multiple imputation (MI) to reduce potential bias due to missingness. Missing values were imputed for covariates with incomplete data. Missing data were assumed to be missing at random (MAR), meaning that the probability of missingness could be explained by observed variables rather than by the unobserved values themselves. Multiple imputation by chained equations (MICE) was applied. Continuous variables were imputed using predictive mean matching, binary variables using logistic regression, and unordered categorical variables using multinomial logistic regression. A total of 20 imputed datasets were generated with 30 iterations. Imputation diagnostics were performed by examining convergence across iterations and by comparing the distributions of observed and imputed values for key variables.

We used multivariable linear regression models to examine the associations between early childhood caries indicators and cognitive outcomes. Regression analyses were performed separately in each imputed dataset, and regression coefficients and standard errors were pooled using Rubin’s rules to obtain final effect estimates, 95% confidence intervals (CIs), and *p*-values.

Four regression models were constructed progressively: Model 1 was adjusted for child’s age in months and sex. Model 2 was further adjusted for maternal age, maternal education level, and annual household income. Model 3 was additionally adjusted for passive smoking duration per day in the past month, preterm birth status, birth weight, breastfeeding duration, and child’s BMI at four years of age. Model 4 was further adjusted for dietary and oral hygiene behaviors, including sweet snack consumption, candy or chocolate consumption, sugar-sweetened fruit drink consumption, carbonated beverage consumption, toothbrushing frequency, and fluoride application frequency. Regression coefficients (*β*), 95% CIs, and *p*-values were reported for each model.

We additionally conducted restricted cubic spline analyses to examine whether the associations between the dmft index and cognitive outcomes deviated from linearity. We used restricted cubic spline (RCS) models with knots placed at the 5th, 35th, 65th, and 95th percentiles of the dmft index to assess potential non-linear relationships between caries indicators and cognitive outcomes. The RCS models allowed us to examine whether these associations deviated from linearity across the distribution of each exposure.

Statistical significance was defined as *p* < 0.05. All analyses were performed using R version 4.4.3 with the “readxl,” “dplyr,” “tidyr,” “openxlsx,” “stringr,” “ggpubr,” “rstatix,” “mice,” “broom,” “ggplot2,” “rms,” “qgcomp,” “gridExtra,” and “Hmisc” packages.

## 3 Results

### 3.1 Characteristics of the study population

Table 1 presents the demographic and oral health characteristics of the study population at 4 years of age in the Shanghai Birth Cohort, which includes 1,099 participants. The mean maternal age at delivery was 29.8 years with a standard deviation of 3.6 years. Boys slightly outnumbered girls, comprising 52% of the children. Most mothers had attained a bachelor’s degree or higher (60%), and 5.5% experienced pre-term births. Breastfeeding status revealed that 41.3% were breastfed for 6 months or more. Additionally, a total of 287 children (26.1%) were exposed to passive smoking daily in the past month, with a mean birth weight of 3381.7 g and a mean BMI of 15.3 at follow-up. In total, 39% of the children were found to have dental caries, with a mean dmft index of 1.6 and a mean number of erupted teeth of 20.1. The mean (SD) scores for FSIQ, VCI, VSI, FRI, WMI, and PSI were 116.0 (12.0), 117.2 (13.3), 114.6 (14.2), 110.5 (12.7), 105.6 (12.5), and 106.5 (11.4), respectively. Children with dental caries had significantly lower mean FSIQ (*p* = 0.005) and VCI scores (*p* = 0.021) compared with caries-free children, while no significant differences were observed in VSI, FRI, WMI, or PSI scores (all *p* > 0.05). Significant group differences were also observed for maternal education level (*p* = 0.049), passive smoking duration (*p* = 0.048), sweetened fruit-flavored beverage intake (*p* = 0.006), and fluoride application frequency (*p* = 0.003).

**Table 1:** Demographic and oral health characteristics of the study population at 4 years of age in the Shanghai Birth Cohort (*n* = 1,099).

Characteristics	Mean ± SD/*N* (%)	Dental caries		*p* value
		No (*n* = 670)	Yes (*n* = 429)	
Child sex				0.226
Boys	572 (52%)	359 (53.6%)	213 (49.7%)	
Girls	527 (48%)	311 (46.4%)	216 (50.3%)	
Child age (months)	53.8 ± 3.3	53.89 ± 3.4	53.58 ± 3.2	0.125
Maternal age at delivery (years)	29.8 ± 3.6	29.82 ± 3.5	29.8 ± 3.7	0.955
Maternal education level				**0.049**
Less than Bachelor’s degree	233 (21.2%)	125 (18.7%)	108 (25.2%)	
Bachelor’s degree	507 (46.1%)	325 (48.5%)	182 (42.4%)	
Graduate level degree	153 (13.9%)	97 (14.5%)	56 (13.1%)	
Missing	206 (18.7%)	123 (18.4%)	83 (19.3%)	
Annual household income				0.060
<50,000	10 (0.9%)	6 (0.9%)	4 (0.9%)	
50,000 ~ 99,999	41 (3.7%)	25 (3.7%)	16 (3.7%)	
100,000 ~ 149,999	108 (9.8%)	64 (9.6%)	44 (10.3%)	
150,000 ~ 299,999	266 (24.2%)	181 (27%)	85 (19.8%)	
≥300,000	95 (8.6%)	63 (9.4%)	32 (7.5%)	
Missing	579 (52.7%)	331 (49.4%)	248 (57.8%)	
Passive smoking duration (hours/day)				**0.048**
Never	812 (73.9%)	517 (77.2%)	295 (68.8%)	
<1 h	140 (12.7%)	74 (11%)	66 (15.4%)	
1–2 h	62 (5.6%)	33 (4.9%)	29 (6.8%)	
3–5 h	35 (3.2%)	19 (2.8%)	16 (3.7%)	
>5 h	50 (4.5%)	27 (4%)	23 (5.4%)	
Preterm birth				0.378
No	968 (88.1%)	583 (87%)	385 (89.7%)	
Yes	60 (5.5%)	39 (5.8%)	21 (4.9%)	
Missing	71 (6.5%)	48 (7.2%)	23 (5.4%)	
Breastfeeding status				
Never breastfed	14 (1.3%)	9 (1.3%)	5 (1.2%)	0.973
< 6 months	165 (15%)	98 (14.6%)	67 (15.6%)	
6 months −12 months	419 (38.1%)	253 (37.8%)	166 (38.7%)	
Breastfed > 12 months	394 (35.9%)	243 (36.3%)	151 (35.2%)	
Missing	107 (9.7%)	67 (10%)	40 (9.3%)	
Birth weight (g)	3381.7 ± 477.4	3362.4 ± 471.3	3411.2 ± 485.6	0.111
BMI	15.3 ± 3.0	15.2 ± 2.3	15.4 ± 3.9	0.312
Sweet pastry				
≤1–3 times /month	100 (9.1%)	58 (8.7%)	42 (9.8%)	0.193
1–3 times /week	369 (33.6%)	241 (36%)	128 (29.8%)	
≥4 times /week	604 (55%)	357 (53.3%)	247 (57.6%)	
Missing	26 (2.4%)	14 (2.1%)	12 (2.8%)	
Candy and chocolate				
≤1–3 times /month	372 (33.8%)	240 (35.8%)	132 (30.8%)	0.175
1–3 times /week	394 (35.9%)	242 (36.1%)	152 (35.4%)	
≥4 times /week	305 (27.8%)	173 (25.8%)	132 (30.8%)	
Missing	28 (2.5%)	15 (2.2%)	13 (3%)	
Sweetened fruit-flavored beverages				
Rarely or never	278 (25.3%)	192 (28.7%)	86 (20%)	**0.006**
1–3 times /month	392 (35.7%)	238 (35.5%)	154 (35.9%)	
≥1 time /week	404 (36.8%)	227 (33.9%)	177 (41.3%)	
Missing	25 (2.3%)	13 (1.9%)	12 (2.8%)	
Carbonated drinks				
Rarely or never	859 (78.2%)	539 (80.4%)	320 (74.6%)	0.113
1–3 times/month	144 (13.1%)	79 (11.8%)	65 (15.2%)	
≥1 time/week	68 (6.2%)	35 (5.2%)	33 (7.7%)	
Missing	28 (2.5%)	17 (2.5%)	11 (2.6%)	
Fluoride application frequency				
Never	422 (38.4%)	284 (42.4%)	138 (32.2%)	**0.003**
1 time	369 (33.6%)	212 (31.6%)	157 (36.6%)	
≥2 times	259 (23.6%)	151 (22.5%)	108 (25.2%)	
Missing	49 (4.5%)	23 (3.4%)	26 (6.1%)	
Toothbrushing frequency				
≤1 time/day	336 (30.6%)	203 (30.3%)	133 (31%)	0.900
>1 time/day	742 (67.5%)	455 (67.9%)	287 (66.9%)	
Missing	21 (1.9%)	12 (1.8%)	9 (2.1%)	
Dental caries				
No	670 (61%)	/	/	
Yes	429 (39%)	/	/	
dmft	1.6 ± 3.1	/	/	/
VCI (verbal comprehension index)	117.2 ± 13.3	118.0 ± 13.0	116.1 ± 13.7	**0.021**
VSI (visual spatial index)	114.6 ± 14.2	115.2 ± 14.4	113.7 ± 13.8	0.087
FRI (fluid reasoning index)	110.5 ± 12.7	111.0 ± 12.3	109.7 ± 13.3	0.083
WMI (working memory index)	105.6 ± 12.5	106.0 ± 12.4	104.9 ± 12.7	0.181
PSI (processing speed index)	106.5 ± 11.4	106.8 ± 11.1	105.9 ± 11.7	0.204
FSIQ (full-scale intelligence quotient)	116.0 ± 12.0	116.8 ± 11.6	114.7 ± 12.5	**0.005**

Bold values indicate statistical significance at *p* < 0.05.

### 3.2 The association between oral health status and child IQ based on linear regression

Table 2 and Supplementary Figure 1 present the adjusted associations between early childhood caries and child IQ, showing that dental caries status was significantly associated with children’s FSIQ in the multivariable analysis. Compared with children without caries, those with caries had lower FSIQ scores (*β* = −1.59; 95% CI: −3.01, −0.18). Additionally, the dmft index was negatively associated with both FSIQ (*β* = −0.25; 95% CI: −0.48, −0.03) and VCI (*β* = −0.31; 95% CI: −0.56, −0.05). No significant associations were observed between dmft index and the other subscale scores, including VSI, FRI, WMI, and PSI.

**Table 2:** Adjusted associations between early childhood caries and child intelligence quotient using linear regression in the Shanghai Birth Cohort (*N* = 1,099).

Early childhood caries indicators	VCI [*β* (95% CI)]				VSI [*β* (95% CI)]				FRI [*β* (95% CI)]			
	Model 1	Model 2	Model 3	Model 4	Model 1	Model 2	Model 3	Model 4	Model 1	Model 2	Model 3	Model 4
Dental caries												
No	Reference											
Yes	**−1.83 (−3.42, −0.24)**	−1.42 (−2.99, 0.14)	−1.38 (−2.95, 0.18)	−1.38 (−2.95, 0.20)	−1.28 (−2.98, 0.42)	−0.92 (−2.61, 0.76)	−0.89 (−2.58, 0.80)	−0.81 (−2.52, 0.89)	−1.20 (−2.73, 0.32)	−0.81 (−2.32, 0.70)	−0.70 (−2.21, 0.81)	−0.58 (−2.11, 0.95)
**dmft**	**−0.35 (−0.61, −0.10)**	**−0.29 (−0.54, −0.04)**	**−0.30 (−0.55, −0.05)**	**−0.31 (−0.56, −0.05)**	−0.24 (−0.52, 0.03)	−0.19 (−0.46, 0.08)	−0.21 (−0.48, 0.06)	−0.20 (−0.48, 0.07)	−0.09 (−0.33, 0.16)	−0.04 (−0.28, 0.21)	−0.03 (−0.27, 0.21)	−0.03 (−0.27, 0.22)

Early childhood caries indicators	WMI [*β* (95% CI)]				PSI [*β* (95% CI)]				FSIQ [*β* (95% CI)]			
	Model 1	Model 2	Model 3	Model 4	Model 1	Model 2	Model 3	Model 4	Model 1	Model 2	Model 3	Model 4
Dental caries												
No												
Yes	−0.96 (−2.46, 0.55)	−0.70 (−2.21, 0.80)	−0.91 (−2.42, 0.61)	−1.02 (−2.55, 0.51)	−1.05 (−2.40, 0.30)	−0.90 (−2.25, 0.45)	−1.01 (−2.37, 0.34)	−1.01 (−2.38, 0.36)	**−2.02 (−3.46, −0.59)**	**−1.60 (−3.01, −0.20)**	**−1.62 (−3.02, −0.21)**	**−1.59 (−3.01, −0.18)**
**dmft**	−0.08 (−0.32, 0.17)	−0.04 (−0.28, 0.20)	−0.07 (−0.32, 0.17)	−0.09 (−0.34, 0.15)	−0.09 (−0.31, 0.13)	−0.07 (−0.29, 0.14)	−0.11 (−0.33, 0.11)	−0.11 (−0.33, 0.11)	**−0.29 (−0.52, −0.06)**	**−0.23 (−0.45, −0.002)**	**−0.25 (−0.47, −0.02)**	**−0.25 (−0.48, −0.03)**

The results of the linear regression models are presented as regression coefficients (β) and 95% confidence intervals (95% CIs). The analytical sample consisted of 1,099 children. Model 1 was adjusted for child’s age in months and child sex. Model 2 was further adjusted for maternal age, maternal education level, and annual household income. Model 3 was further adjusted for passive smoking, preterm birth, birth weight, breastfeeding status, and body mass index (BMI). Model 4 was further adjusted for sweet pastry, candy and chocolate, sweetened fruit-flavored beverages, carbonated drinks, fluoride application frequency, and toothbrushing frequency. Bold values indicate statistical significance at *p* < 0.05.

### 3.3 Restricted cubic spline analyses

**Figure 3:**
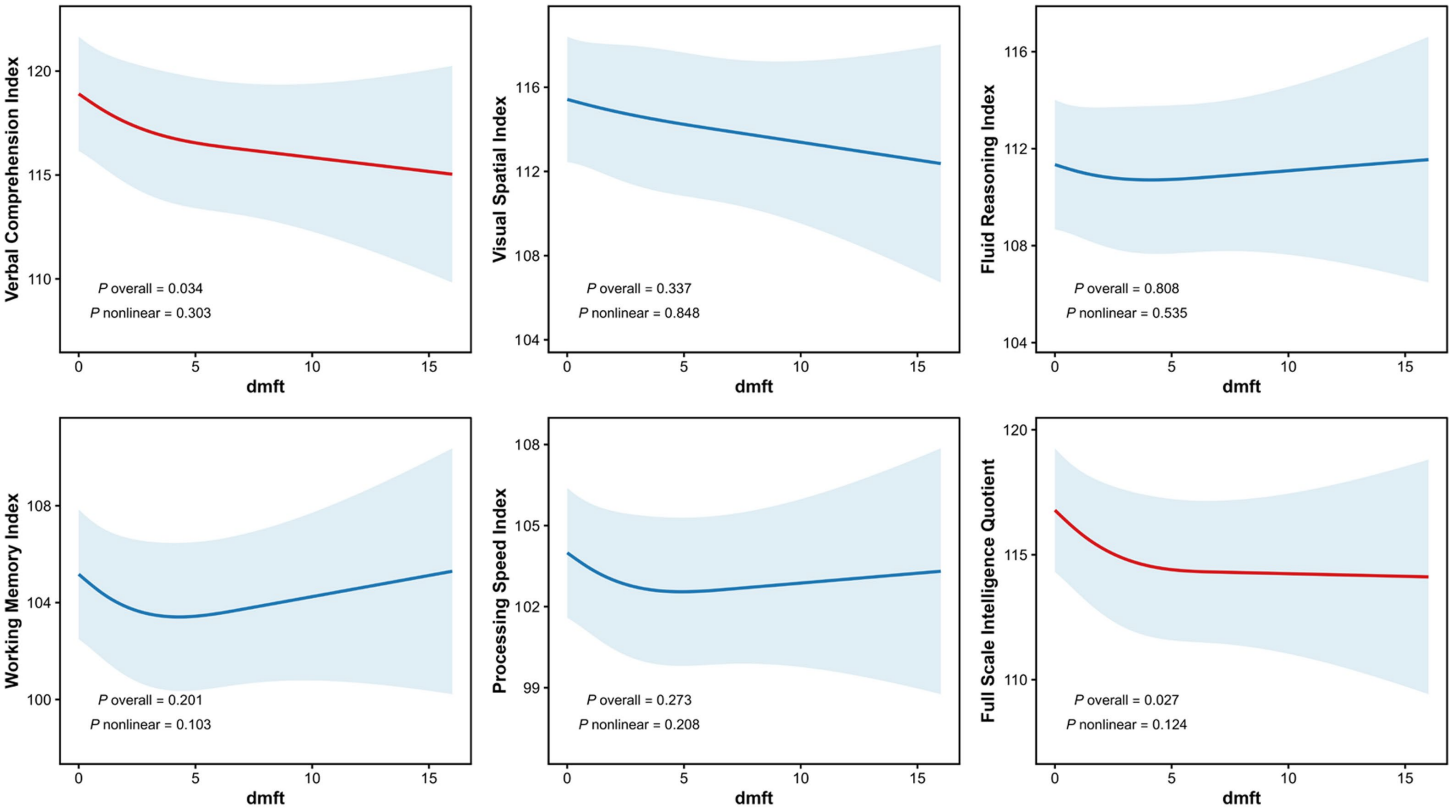
Restricted cubic spline curves for the associations between the dmft index and child cognitive outcomes. This figure shows the dose–response relationships between the decayed, missing, and filled teeth (dmft) index and various cognitive outcomes, estimated using restricted cubic spline (RCS) models after multiple imputation. The solid lines represent the adjusted predicted cognitive scores, and the shaded areas indicate the corresponding 95% confidence intervals (CIs). The curves were plotted over the 1st–99th percentile range of dmft. The models were adjusted for child’s age, sex, maternal age, maternal education, household income, passive smoking exposure, body mass index, breastfeeding duration, prematurity, birth weight, sweet food intake, sugar-sweetened beverage intake, toothbrushing frequency, and fluoride application.

The RCS analyses indicated that the associations between the dmft index and various domains of cognitive function generally did not deviate significantly from linearity, as illustrated in Figure 3. All *p* values for non-linearity were > 0.05, indicating no statistically significant evidence of non-linear associations. Significant overall associations were observed for VCI (*P*_overall_ = 0.034) and FSIQ (*P*_overall_ = 0.027), with higher dmft values generally associated with lower predicted scores. The observed patterns for VCI and FSIQ were approximately linear, with no clear evidence of a threshold effect.

## 4 Discussion

In this cross-sectional study of 1,099 4-year-old children from the Shanghai Birth Cohort, we examined the associations of early childhood caries indicators, including dmft index and caries status, with full-scale and domain-specific IQ scores. We found that children with caries had lower FSIQ scores, and that a higher dmft index was associated with lower FSIQ and VCI scores after adjustment for demographic, socioeconomic, perinatal, dietary, and oral hygiene-related factors. To our knowledge, this is the first study in China to examine the association between early childhood caries (ECC) and cognitive development in preschool children.

### 4.1 Dental caries and child IQ

Previous studies on early childhood caries have increasingly suggested that its impact may extend beyond oral health, with implications for children’s quality of life and overall development (7–19). Although evidence regarding anthropometric growth indicators such as height, weight, and BMI remains inconsistent, cohort studies have reported that early childhood caries may be associated with slower gains in height and weight or subsequent changes in BMI, supporting the possibility that untreated caries may have broader developmental implications (11, 46–50). Physical growth and cognitive development are closely interrelated during early childhood, a period characterized by rapid brain development and high neurodevelopmental plasticity (20–22). Therefore, caries-related pain, impaired mastication, sleep disturbance, nutritional problems, and systemic inflammation may also be relevant to cognitive development in preschool children (7–19, 51, 52).

Compared with previous studies that mainly focused on physical growth, school absenteeism, or academic performance (25–36), our study focused on preschool children and used the WPPSI-IV to assess multidimensional intelligence profiles before formal schooling begins. This is important because cognitive abilities in early childhood, including executive control, memory, reasoning, and language-related abilities, are foundational for later learning and school adaptation (20–24). Our findings that caries status was associated with lower FSIQ and that a higher dmft index was associated with lower FSIQ and VCI provide preliminary evidence that oral health may be linked to early cognitive development in the preschool period.

Previous studies have also examined the relationship between developmental or cognitive status and oral health, often treating developmental delay or IQ as the exposure and dental caries as the outcome (53–55). In a study of low-income preschool children in the United States, children with developmental delays had a higher prevalence of dental caries than those without developmental delays (37). Studies among school-aged children have also reported a higher caries burden among children with lower IQ, although some associations were not statistically significant, possibly due to limited sample size, age differences, or incomplete control for confounding (54, 55). These findings are broadly consistent with our results and suggest that oral health and cognitive development may be closely interrelated (53–55). In the present study, direct measures of academic performance were unavailable due to the young age of participants—four-year-old preschool children. Instead, we employed the WPPSI-IV to assess intelligence, which provides a more direct, accurate, and comprehensive evaluation of children’s cognitive abilities than academic performance. These intelligence scores serve as early cognitive indicators that can better predict future learning potential and academic achievement. Our findings align with previous studies reporting a significant association between children’s oral health and school performance. For instance, a large, nationally representative study in the United States involving 41,988 children aged 6 to 17 years found that poor oral health was significantly associated with decreased school performance (25). Similarly, another study of 45,711 children aged 6–17 in the 2016–2017 National Survey of Children’s Health showed that children’s oral health is still closely related to their academic performance (26).

Several plausible pathways may be involved in the observed association between early childhood caries and child IQ. First, severe early childhood caries is often accompanied by pain, discomfort, and infection, which may disturb sleep, reduce attention, and interfere with daily functioning, thereby potentially relating to cognitive performance in young children (7, 11, 14, 17–19, 56). Second, untreated caries may impair mastication and food intake. Reduced mastication has been linked to poorer memory-related functions in previous studies, and inadequate food intake may contribute to nutritional problems during a sensitive period of growth and neurodevelopment (16, 57–61). Third, poor oral health may coexist with broader behavioral and family-level factors, such as dietary habits, oral hygiene practices, and access to preventive dental care, which may also be related to children’s cognitive development. However, this association should not be interpreted as evidence of a causal effect. The possibility of reverse causality should also be considered as children with lower cognitive performance may have greater difficulty maintaining regular oral hygiene behaviors, cooperating with dental care, or regulating cariogenic dietary habits, which could increase their risk of dental caries (53–55). Therefore, the relationship between early childhood caries and child IQ may be bidirectional rather than strictly unidirectional. Given the cross-sectional design of the present study, the temporal sequence between dental caries and cognitive performance cannot be determined, and these mechanisms should be interpreted cautiously.

Although the effect sizes observed in this study were small at the individual level, they are more appropriately interpreted from a population health perspective than a clinical one. Early childhood caries is common among preschool children, including in our study population, and occurs during a period of rapid brain development. In this context, even small differences in average cognitive scores can be relevant at the population level when a large proportion of children are exposed. Our findings do not imply that dental caries alone determines cognitive outcomes in individual children; instead, they suggest that oral health may represent one of several modifiable early-life factors associated with cognitive development. From a public health standpoint, these results point to the potential value of incorporating early oral health promotion and caries prevention into broader child health and development strategies.

### 4.2 Strengths and limitations

This study is among the first to systematically investigate the association between caries and intelligence development among preschool children in Shanghai, demonstrating strong novelty and regional relevance. Our study has several strengths. First, all our measurements were conducted by professional clinicians, ensuring the accuracy and validity of the data. Additionally, we assessed children’s IQ during the preschool period using the most recent edition of the Wechsler Preschool and Primary Scale of Intelligence (WPPSI-IV), a widely recognized cognitive assessment tool known for its strong psychometric validity and reliability (62). Second, the use of progressively adjusted models allowed us to account for a wide range of potential confounders, including demographic, socioeconomic, perinatal, health-related, dietary, and oral hygiene factors. Third, the participants in this study were drawn from a rigorously designed birth cohort that prospectively collected a large amount of information on relevant covariates during pregnancy and conducted regular follow-ups on children’s health, enabling us to adjust for multiple potential confounding factors (39).

However, several limitations should be noted. First, the cross-sectional design precludes any inference of causality, as dental caries and cognitive performance were assessed at the same time point, the temporal direction of the observed association cannot be established, and the possibility of reverse causality cannot be fully excluded. Future longitudinal studies are needed to confirm the directionality of these associations. Second, the study sample was restricted to 4-year-old children, and due to their young age, academic performance data were unavailable; thus, intelligence scores were used as indicators of cognitive development, limiting the generalizability of findings to other age groups or long-term outcomes. Third, although the study was embedded within a well-characterized birth cohort with prospectively collected maternal and child health data, the analytic sample consisted predominantly of families with relatively high educational attainment. Therefore, caution is needed when extrapolating these findings to children from rural areas, other regions of China, or populations with lower socioeconomic status. Finally, although we adjusted for a range of sociodemographic, perinatal, dietary, and oral hygiene-related factors, residual confounding cannot be fully excluded. Important determinants of child cognitive development, such as parental cognitive ability, the quality of the home learning environment, and detailed nutritional factors, were not available in the present analysis.

## 5 Conclusion

This study is among the first to investigate the associations between early childhood caries and cognitive function in preschool children using multidimensional intelligence assessments. We found that higher levels of dental caries, especially as measured by the dmft index, were associated with lower IQ scores after accounting for demographic, socioeconomic, perinatal, health-related, dietary, and oral hygiene factors. These findings should be interpreted as population-level associations rather than deterministic outcomes for individual children. Given the cross-sectional design, the temporal sequence of the observed associations could not be established, and the possibility of reverse causality cannot be fully excluded. Further longitudinal studies are warranted to confirm these findings and clarify the underlying mechanisms.

## Data Availability

The datasets analyzed in this study are not publicly available because they contain sensitive information from children and their families and are subject to privacy and ethical restrictions. Access to the datasets requires approval from the relevant ethics committee and study investigators. Requests to access these datasets should be directed to HZ, haozhang_99@fudan.edu.cn.

## Footnotes

1
https://dagitty.net/

## Supplementary material

SUPPLEMENTARY FIGURE 1 Forest plot displaying the associations between individual oral health indicators and child IQ outcomes, based on multivariable linear regression models. This figure shows the regression coefficients (*β*) and corresponding 95% confidence intervals (CIs) for the associations between oral health indicators and child intelligence outcomes. All models were adjusted for child age in months, child sex, maternal age, maternal education level, household income, passive smoking exposure, preterm birth, birth weight, breastfeeding duration, body mass index, frequency of sweet food intake, frequency of sugar-sweetened beverage intake, toothbrushing frequency, and fluoride application frequency. A negative *β* value indicates lower IQ scores associated with poorer oral health status.

## References

[1] Lam PPY Chua H Ekambaram M Lo ECM Yiu CKY. Does early childhood caries increase caries development among school children and adolescents? A systematic review and meta-analysis. Int J Environ Res Public Health. (2022) 19:13459. doi: 10.3390/ijerph192013459, 36294037 PMC9603429

[2] Jiang H f Shi A t Li J Zhang YH Yang J. Effectiveness of risk-based caries management among Chinese preschool children: a randomized controlled single-blind trial. BMC Oral Health. (2024) 24:673. doi: 10.1186/s12903-024-04442-z, 38851679 PMC11162041

[3] Uribe SE Innes N Maldupa I. The global prevalence of early childhood caries: a systematic review with meta-analysis using the WHO diagnostic criteria. Int J Paediatr Dent. (2021) 31:817–30. doi: 10.1111/ipd.12783, 33735529

[4] Gupta M Rao BD Shah K Narang GS. Does serum ferritin level affect early childhood caries?-a review. Int J Clin Pediatr Dent. (2025) 18:487–90. doi: 10.5005/jp-journals-10005-3116 40469822 PMC12131059

[5] Maklennan A Borg-Bartolo R Wierichs RJ Esteves-Oliveira M Campus G. A systematic review and meta-analysis on early-childhood-caries global data. BMC Oral Health. (2024) 24:835. doi: 10.1186/s12903-024-04605-y, 39049051 PMC11267837

[6] Dhull KS Dutta B Pattanaik S Gupta A Indira MD Wandile B. Decoding early childhood caries: a comprehensive review navigating the impact of evolving dietary trends in preschoolers. Cureus. (2024) 16:e58170. doi: 10.7759/cureus.58170 38741840 PMC11090680

[7] Zaror C Matamala-Santander A Ferrer M Rivera‐Mendoza F Espinoza‐Espinoza G Martínez‐Zapata MJ. Impact of early childhood caries on oral health-related quality of life: a systematic review and meta-analysis. Int J Dent Hyg. (2022) 20:120–35. doi: 10.1111/idh.12494, 33825317

[8] Setiawan AS Indriyanti R Suryanti N Rahayuwati L Juniarti N. Neonatal stunting and early childhood caries: a mini-review. Front Pediatr. (2022) 10:871862. doi: 10.3389/fped.2022.871862, 35923789 PMC9339654

[9] Meyer F Enax J. Early childhood caries: epidemiology, aetiology, and prevention. Int J Dent. (2018) 2018:1415873. doi: 10.1155/2018/1415873, 29951094 PMC5987323

[10] Naidu R Nunn J Donnelly-Swift E. Oral health-related quality of life and early childhood caries among preschool children in Trinidad. BMC Oral Health. (2016) 16:128. doi: 10.1186/s12903-016-0324-7 27923355 PMC5142136

[11] Sheiham A. Dental caries affects body weight, growth and quality of life in pre-school children. Br Dent J. (2006) 201:625–6. doi: 10.1038/sj.bdj.4814259 17128231

[12] Gussy MG Waters EG Walsh O Kilpatrick NM. Early childhood caries: current evidence for aetiology and prevention. J Paediatr Child Health. (2006) 42:37–43. doi: 10.1111/j.1440-1754.2006.00777.x, 16487388

[13] Souto-Souza D Soares MEC Primo-Miranda EF Pereira LJ Ramos-Jorge ML Ramos-Jorge J. The influence of malocclusion, sucking habits and dental caries in the masticatory function of preschool children. Braz Oral Res. (2020) 34:e059. doi: 10.1590/1807-3107bor-2020.vol34.0059 32578802

[14] Collado V Pichot H Delfosse C Eschevins C Nicolas E Hennequin M. Impact of early childhood caries and its treatment under general anesthesia on orofacial function and quality of life: a prospective comparative study. Med Oral Patol Oral Cir Bucal. (2017) 22:e333–41. doi: 10.4317/medoral.21611, 28390125 PMC5432082

[15] Gavião MB Raymundo VG Sobrinho LC. Masticatory efficiency in children with primary dentition. Pediatr Dent. (2001) 23:499–505. 11800451

[16] Fukushima-Nakayama Y Ono T Hayashi M Inoue M Wake H Nakashima T. Reduced mastication impairs memory function. J Dent Res. (2017) 96:1058–66. doi: 10.1177/0022034517708771, 28621563

[17] Fallone G Acebo C Arnedt JT Seifer R Carskadon MA. Effects of acute sleep restriction on behavior, sustained attention, and response inhibition in children. Percept Mot Skills. (2001) 93:213–29. doi: 10.2466/pms.2001.93.1.213 11693688

[18] Diaz A Berger R Valiente C Eisenberg N VanSchyndel SK Tao C . Children’s sleep and academic achievement: the moderating role of effortful control. Int J Behav Dev. (2017) 41:275–84. doi: 10.1177/0165025416635284, 28255190 PMC5327793

[19] Stormark KM Fosse HE Pallesen S Hysing M. The association between sleep problems and academic performance in primary school-aged children: findings from a norwegian longitudinal population-based study. PLoS One. (2019) 14:e0224139. doi: 10.1371/journal.pone.0224139, 31697711 PMC6837329

[20] Lenroot RK Giedd JN. Brain development in children and adolescents: insights from anatomical magnetic resonance imaging. Neurosci Biobehav Rev. (2006) 30:718–29. doi: 10.1016/j.neubiorev.2006.06.001, 16887188

[21] Goldman-Rakic PS. Development of cortical circuitry and cognitive function. Child Dev. (1987) 58:601–22. doi: 10.2307/1130201 3608641

[22] Riggins T Geng F Botdorf M Canada K Cox L Hancock GR. Protracted hippocampal development is associated with age-related improvements in memory during early childhood. NeuroImage. (2018) 174:127–37. doi: 10.1016/j.neuroimage.2018.03.009, 29518573 PMC5949262

[23] Blair C Razza RP. Relating effortful control, executive function, and false belief understanding to emerging math and literacy ability in kindergarten. Child Dev. (2007) 78:647–63. doi: 10.1111/j.1467-8624.2007.01019.x, 17381795

[24] Alloway TP Alloway RG. Investigating the predictive roles of working memory and IQ in academic attainment. J Exp Child Psychol. (2010) 106:20–9. doi: 10.1016/j.jecp.2009.11.003, 20018296

[25] Guarnizo-Herreño CC Wehby GL. Children’s dental health, school performance and psychosocial well-being. J Pediatr. (2012) 161:1153–1159.e2. doi: 10.1016/j.jpeds.2012.05.025, 22727866 PMC3459270

[26] Guarnizo-Herreño CC Lyu W Wehby GL. Children’s oral health and academic performance: evidence of a persisting relationship over the last decade in the United States. J Pediatr. (2019) 209:183–189.e2. doi: 10.1016/j.jpeds.2019.01.045, 30926152 PMC6667186

[27] Quadri MFA Ahmad B. The mediation pathway linking dental caries and academic performance in children. Caries Res. (2025) 59:1–10. doi: 10.1159/000540883, 39137743

[28] Seirawan H Faust S Mulligan R. The impact of oral health on the academic performance of disadvantaged children. Am J Public Health. (2012) 102:1729–34. doi: 10.2105/AJPH.2011.300478, 22813093 PMC3482021

[29] Paula JS Lisboa CM de Castro Meneghim M Pereira AC Ambrosano GMB Mialhe FL. School performance and oral health conditions: analysis of the impact mediated by socio-economic factors. Int J Paediatr Dent. (2016) 26:52–9. doi: 10.1111/ipd.12158 25752583

[30] Slade GD Sanders A. Two decades of persisting income-disparities in dental caries among U.S. children and adolescents. J Public Health Dent. (2018) 78:187–91. doi: 10.1111/jphd.12261, 29243816 PMC6003830

[31] Dye BA Mitnik GL Iafolla TJ Vargas CM. Trends in dental caries in children and adolescents according to poverty status in the United States from 1999 through 2004 and from 2011 through 2014. J Am Dent Assoc. (2017) 148:550–565.e7. doi: 10.1016/j.adaj.2017.04.013, 28619207

[32] Pongpichit B Sheiham A Pikhart H Tsakos G. Time absent from school due to dental conditions and dental care in thai schoolchildren. J Public Health Dent. (2008) 68:76–81. doi: 10.1111/j.1752-7325.2007.00051.x 18661602

[33] Garg N Anandakrishna L Chandra P. Is there an association between oral health status and school performance? A preliminary study. Int J Clin Pediatr Dent. (2012) 5:132–5. doi: 10.5005/jp-journals-10005-1150, 25206152 PMC4148740

[34] El-Sayed M Osman K Nour A. Prevalence of dental caries and its impact on the academic performance of Sudanese basic school children, Al-sahafa residential area (2013-2014). J Am Sci. (2015) 11:195–203.

[35] Almeida RF Leal SC Medonca JGA Hilgert LA Ribeiro APD. Oral health and school performance in a group of schoolchildren from the federal district, Brazil. J Public Health Dent. (2018) 78:306–12. doi: 10.1111/jphd.12273 29752807

[36] Piovesan C Antunes JLF Mendes FM Antunes JL Guedes RS Ardenghi TM. Influence of children’s oral health-related quality of life on school performance and school absenteeism. J Public Health Dent. (2012) 72:156–63. doi: 10.1111/j.1752-7325.2011.00301.x, 22372974

[37] Chi DL Rossitch KC Beeles EM. Developmental delays and dental caries in low-income preschoolers in the USA: a pilot cross-sectional study and preliminary explanatory model. BMC Oral Health. (2013) 13:53. doi: 10.1186/1472-6831-13-53, 24119240 PMC3906997

[38] Du RY Yiu CK King NM Wong VC McGrath CP. Oral health among preschool children with autism spectrum disorders: a case-control study. Autism. (2015) 19:746–51. doi: 10.1177/1362361314553439, 25432504

[39] Zhang J Tian Y Wang W Ouyang F Xu J Yu X . Cohort profile: the Shanghai birth cohort. Int J Epidemiol. (2019) 48:21–21g. doi: 10.1093/ije/dyy277, 30629180

[40] Rai A Sundas S Dhakal N Khapung A. Assessment of dental caries based on ICDAS and WHO criteria: a comparative study. Int J Paediatr Dent. (2024) 34:77–84. doi: 10.1111/ipd.13099 37330985

[41] Park SE Demakis GJ. "Wechsler preschool and primary scale of intelligence". In: Eds. Zeigler-Hill V, Shackelford T. Encyclopedia of Personality and Individual Differences. Cham: Springer (2017). p. 1–4. doi: 10.1007/978-3-319-28099-8_1037-1

[42] Zhang J Deng H Huang X Wang L Zhou P Zeng J . Pre-school children single inhalation anesthetic exposure and neuro-psychological development: a prospective study and Mendelian randomization analysis. Front Neurol. (2024) 15:1389203. doi: 10.3389/fneur.2024.1389203 38933327 PMC11199877

[43] Tong J Liang C Wu X Huang K Zhu B Gao H . Prenatal serum thallium exposure and cognitive development among preschool-aged children: a prospective cohort study in China. Environ Pollut. (2022) 293:118545. doi: 10.1016/j.envpol.2021.118545, 34801620

[44] Ramsden S Richardson FM Josse G Thomas MSC Ellis C Shakeshaft C . Verbal and nonverbal intelligence changes in the teenage brain. Nature. (2011) 479:113–6. doi: 10.1038/nature10514, 22012265 PMC3672949

[45] Wang H Luo F Zhang Y Yang X Zhang S Zhang J . Prenatal exposure to perfluoroalkyl substances and child intelligence quotient: evidence from the Shanghai birth cohort. Environ Int. (2023) 174:107912. doi: 10.1016/j.envint.2023.107912, 37023630

[46] Gunay B Kaya MS Ozgen IT Guler EM Kocyigit A. Evaluation of the relationship between pain inflammation due to dental caries and growth parameters in preschool children. Clin Oral Investig. (2023) 27:3721–30. doi: 10.1007/s00784-023-04988-2, 37036512 PMC10088690

[47] MCM v G-S Amerongen EW Aartman IHA Wennink JMB ten Cate JM de Soet JJ. The influence of dental caries on body growth in prepubertal children. Clin Oral Investig. (2011) 15:141–9. doi: 10.1007/s00784-010-0380-3, 20111879

[48] Turton B Chher T Hak S Sokal-Gutierrez K Lopez Peralta D Lailou A . Associations between dental caries and ponderal growth in children: a cambodian study. J Glob Health. (2025) 12:4046. doi: 10.7189/jogh.12.04046 PMC9204672 35713031

[49] Kay EJ Northstone K Ness A Duncan K Crean SJ. Is there a relationship between birthweight and subsequent growth on the development of dental caries at 5 years of age? A cohort study. Community Dent Oral Epidemiol. (2010) 38:408–14. doi: 10.1111/j.1600-0528.2010.00548.x, 20545719

[50] Lempert SM Froberg K Christensen LB Kristensen PL Heitmann BL. Association between body mass index and caries among children and adolescents. Community Dent Oral Epidemiol. (2014) 42:53–60. doi: 10.1111/cdoe.12055, 23763718

[51] Bagis EE Derelioglu SS Sengül F Yılmaz S. The effect of the treatment of severe early childhood caries on growth-development and quality of life. Children. (2023) 10:411. doi: 10.3390/children10020411, 36832541 PMC9955375

[52] Aung YM Jelleyman T Ameratunga S Tin Tin S. Body mass index and dental caries in New Zealand pre-school children: a population-based study. J Paediatr Child Health. (2021) 57:1432–7. doi: 10.1111/jpc.15500 33860964

[53] Thomson W Broadbent J Caspi A Thomson WM Broadbent JM Poulton R . Childhood IQ predicts age-38 oral disease experience and service-use. Community Dent Oral Epidemiol. (2019) 47:252–8. doi: 10.1111/cdoe.12451, 30812053 PMC6520161

[54] Dhanu G Havale R Shrutha SP Quazi N Shafna TP Ahemd A. Assessment of intelligence quotient using raven’s coloured progressive matrices among school children of Hyderabad Karnataka region and its correlation with prevalence of dental caries. J Indian Soc Pedod Prev Dent. (2019) 37:25–30. doi: 10.4103/JISPPD.JISPPD_236_18, 30804304

[55] Navit S Malhotra G Singh J Naresh V Anshul PN. Interrelationship of intelligence quotient with caries and gingivitis. J Int Oral Health. (2014) 6:56–62. PMC4148575 25214734

[56] McGrath C Broder H Wilson-Genderson M. Assessing the impact of oral health on the life quality of children: implications for research and practice. Comm Dent Oral Epidemiol. (2004) 32:81–5. doi: 10.1111/j.1600-0528.2004.00149.x, 15061856

[57] Pierce A Singh S Lee J Grant C de Cruz Jesus V Schroth RJ. The burden of early childhood caries in Canadian children and associated risk factors. Front Public Health. (2019) 7:328. doi: 10.3389/fpubh.2019.00328 31781530 PMC6861386

[58] Aulia RN Indriyanti R Setiawan AS. The bi-directional relationship between growth stunting and early childhood caries: a rapid review. Front Public Health. (2023) 11:1234893. doi: 10.3389/fpubh.2023.1234893, 38146474 PMC10749356

[59] Heinrich-Weltzien R Monse B Benzian H Heinrich J Kromeyer-Hauschild K. Association of dental caries and weight status in 6- to 7-year-old Filipino children. Clin Oral Investig. (2013) 17:1515–23. doi: 10.1007/s00784-012-0849-3 23053701

[60] Kagihara LE Niederhauser VP Stark M. Assessment, management, and prevention of early childhood caries. J Am Acad Nurse Pract. (2009) 21:1–10. doi: 10.1111/j.1745-7599.2008.00367.x, 19125889

[61] Sheetal A Hiremath VK Patil AG Sajjansetty S Kumar SR. Malnutrition and its oral outcome – a review. J Clin Diagn Res. (2013) 7:178–80. doi: 10.7860/JCDR/2012/5104.2702 23449967 PMC3576783

[62] Maselko J Sikander S Bhalotra S Bangash O Ganga N Mukherjee S . Effect of an early perinatal depression intervention on long-term child development outcomes: follow-up of the thinking healthy programme randomised controlled trial. Lancet Psychiatry. (2015) 2:609–17. doi: 10.1016/S2215-0366(15)00109-1 26303558

