# Supplementary figures and images for: Associations of early childhood caries and child intelligence quotient: evidence from the Shanghai Birth Cohort
Source: Front Public Health. 2026 Jun 1;14:1803559. doi: 10.3389/fpubh.2026.1803559 (PMC13265527; doi:10.3389/fpubh.2026.1803559)

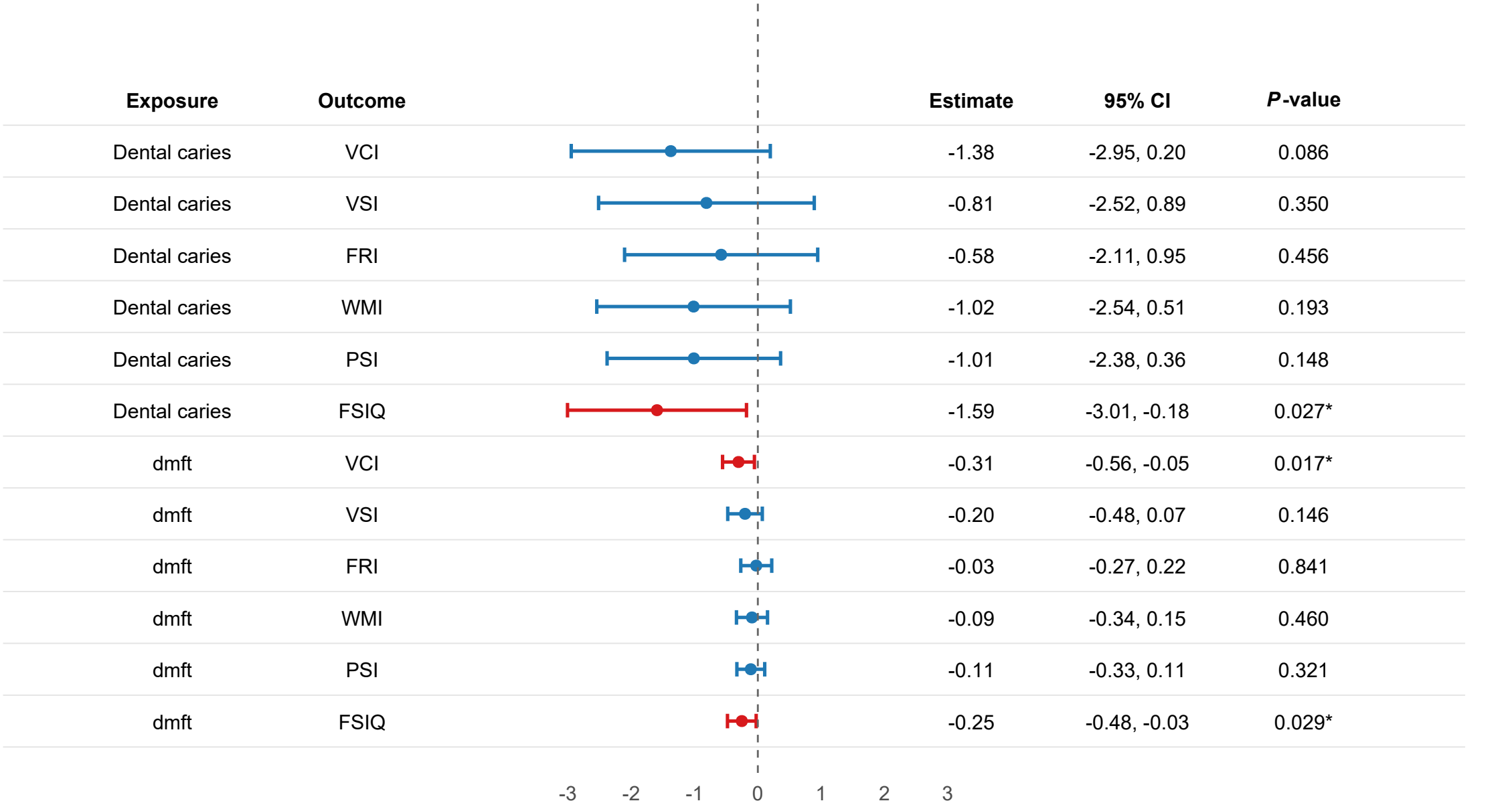

Supplement: SUPPLEMENTARY FIGURE 1 — Forest plot displaying the associations between individual oral health indicators and child IQ outcomes, based on multivariable linear regression models. This figure shows the regression coefficients (β) and corresponding 95% confidence intervals (CIs) for the associations between oral health indicators and child intelligence outcomes. All models were adjusted for child age in months, child sex, maternal age, maternal education level, household income, passive smoking exposure, preterm birth, birth weight, breastfeeding duration, body mass index, frequency of sweet food intake, frequency of sugar-sweetened beverage intake, toothbrushing frequency, and fluoride application frequency. A negative β value indicates lower IQ scores associated with poorer oral health status. [file Image_1.pdf]
